# Supplementary material for: A tumor focused approach to resolving the etiology of DNA mismatch repair deficient tumors classified as suspected Lynch syndrome
Source: J Transl Med. 2023 Apr 26;21:282. doi: 10.1186/s12967-023-04143-1 (PMC10134620; doi:10.1186/s12967-023-04143-1)
Supplement: Supplementary file 1 — Additional file 1: Table S1. Table displaying optimal cut-offs for the six tumor features determined previously (Walker et al. 2023) in the additive feature combination approach. Table S2. SLS tumors (n=13) that showed discordant MMR IHC findings between clinical diagnostic testing before study entry and testing completed internally during this study and the change in their MMR status and/or pattern of MMR protein loss. Table S3. The concordance between the final MMR IHC result and the predicted dMMR status from the additive feature combination approach overall and by tumor type. Table S4. The tumor MLH1 methylation testing completed for SLS tumors prior to entering the study showing either negative, inconclusive, or not tested results and the subsequent MLH1 methylation testing results from internal testing using MethyLight and MS-HRM assays highlighting the positive MLH1 methylation results found by this study. Table S5. Presentation of germline pathogenic variants and variants of uncertain clinical significance (VUS) identified in the MMR, MUTYH and POLE genes. Table S6. Summary of the clinicopathological features for the double somatic MMR mutation (dMMR-DS) tumors overall and by tumor type. Figure S1. Bar plots presenting the results from the additive tumor feature combination approach to assess the MMR status in the double somatic mutation cohort for A) all tumors combined and separated by B) CRC, C) EC and D) SST tissue types. Figure S2. Bar plot presenting the prevalence of pathogenic/likely pathogenic somatic mutations (including loss of heterozygosity, LOH) by subtype for the study cohort. Figure S3. Pie graphs displaying the frequency of the mutation combination type (two single somatic mutations versus a single somatic mutation with loss of heterozygosity (LOH)) as well as the type of mutation A) overall and B) separated by tissue type. Figure S4. Bar graphs presenting the site distribution in the double somatic mutation cohort across all CRCs and SSTs. [file 12967_2023_4143_MOESM1_ESM.docx]

**Additional Files for**

**A tumor focused approach to resolving the etiology of DNA mismatch repair deficient tumors classified as suspected Lynch syndrome**

**Corresponding author:**

Associate Professor Daniel D. Buchanan

Head, Colorectal Oncogenomics Group

Department of Clinical Pathology

The University of Melbourne

Victorian Comprehensive Cancer Centre

305 Grattan Street

Parkville, Victoria, 3010 Australia

Ph: +61 385597004

Email: [daniel.buchanan@unimelb.edu.au](mailto:daniel.buchanan@unimelb.edu.au)

Table of Contents

[**SUPPLEMENTARY METHODS** 4](#_Toc132233519)

[Pathology Characterization 4](#_Toc132233520)

[DNA Mismatch Repair Protein Immunohistochemistry 4](#_Toc132233521)

[DNA Extraction from Tumor and Blood Biospecimen 5](#_Toc132233522)

[Targeted Multi-Gene Panel Testing 5](#_Toc132233523)

[Bioinformatic Analysis 6](#_Toc132233524)

[Determination of DNA Mismatch Repair Status Using the Additive Feature Combination Approach 8](#_Toc132233525)

[Categorization of SLS Cases Using the Accumulated Molecular Results 8](#_Toc132233526)

[PREMM5: Lynch Syndrome Prediction Model 9](#_Toc132233527)

[References 10](#_Toc132233528)

[**SUPPLEMENTARY TABLES** 17](#_Toc132233529)

[**Supplementary Table 1.** Table displaying optimal cut-offs for the six tumor features determined previously (Walker et al., 2022) in the additive feature combination approach. 17](#_Toc132233530)

[**Supplementary Table 2.** SLS tumors (n=13) that showed discordant MMR IHC findings between clinical diagnostic testing before study entry and testing completed internally during this study and the change in their MMR status and/or pattern of MMR protein loss. 18](#_Toc132233531)

[**Supplementary Table 3.** The concordance between the final MMR IHC result and the predicted dMMR status from the additive feature combination approach overall and by tumor type. 20](#_Toc132233532)

[**Supplementary Table 4.** The tumor MLH1 methylation testing completed for SLS tumors prior to entering the study showing either negative, inconclusive, or not tested results and the subsequent MLH1 methylation testing results from internal testing using MethyLight and MS-HRM assays highlighting the positive MLH1 methylation results found by this study. 21](#_Toc132233533)

[**Supplementary Table 5.** Presentation of germline pathogenic variants and variants of uncertain clinical significance (VUS) identified in the MMR, MUTYH and POLE genes. 23](#_Toc132233534)

[**Supplementary Table 6.** Summary of the clinicopathological features for the double somatic MMR mutation (dMMR-DS) tumors overall and by tumor type. 25](#_Toc132233535)

[**SUPPLEMENTARY FIGURES** 26](#_Toc132233536)

[**Supplementary Figure 1. Bar plots presenting the results from the additive tumor feature combination approach to assess the MMR status in the double somatic mutation cohort for A) all tumors combined and separated by B) CRC, C) EC and D) SST tissue types.** 27](#_Toc132233537)

[**Supplementary Figure 2: Bar plot presenting the prevalence of pathogenic / likely pathogenic somatic mutations (including loss of heterozygosity, LOH) by subtype for the study cohort.** 28](#_Toc132233538)

[**Supplementary Figure 3: Pie graphs displaying the frequency of the mutation combination type (two single somatic mutations versus a single somatic mutation with loss of heterozygosity (LOH)) as well as the type of mutation A) overall and B) separated by tissue type.** 30](#_Toc132233539)

[**Supplementary Figure 4: Bar graphs presenting the site distribution in the double somatic mutation cohort across all CRCs and SSTs.** 31](#_Toc132233540)

[**Supplementary Figure 5: Boxplots presenting the site distribution in the double somatic mutation cohort across all A) CRCs and B) SSTs. Significant (< 0.05) p-values are indicated for pairwise (t-test) and multigroup comparisons (Anova).** 32](#_Toc132233541)

[**Supplementary Figure 6: Scatter plots presenting the PREMM5 score distribution in the test cohort for A) all tumors combined and separated by B) CRC, C) EC and D) SST tissue types.** 33](#_Toc132233542)

[**Supplementary Figure 7: The distribution of tumor values for each of the six features that are included in the additive feature combination approach for determining tumor dMMR status grouped by molecular subtype and by combining sporadic dMMR groups dMMR-DS and dMMR-MLH1me into a “sporadic combined” group.** 34](#_Toc132233543)

## **Additional file methods**

## *Pathology Characterization*

For each tumor, pathology reports and Hematoxylin and Eosin (H&E) slides were reviewed by a Pathologist (CR and SD) to determine tumor primary site, tumor grade, histological type, FIGO (International Federation of Gynecology and Obstetrics) stage and sebaceous skin lesion type. In instances where data was missing, tumors were re-staged according to the FIGO 2009 criteria (Pecorelli, 2009).

## *DNA Mismatch Repair Protein Immunohistochemistry*

DNA mismatch repair (MMR) immunohistochemistry (IHC) testing to categorize the tumor as MMR-deficient (dMMR) as part of the SLS diagnosis was performed by various diagnostic pathology services across Australia and New Zealand prior to study. For the study and where tissue was available, MMR IHC was repeated. Briefly, this testing included staining of 4µm sections on a Ventana *DISCOVERY ULTRA* automated stainer (Ventana Medical Systems Inc., Oro Valley, United States) using anti-MLH1 (M1), anti-MSH2 (G219-1129), anti-MSH6 (SP93) mouse monoclonal and anti-PMS2 (A16-4) rabbit monoclonal primary antibodies (Roche Diagnostics, Basel, Switzerland). All staining protocols were performed according to manufacturer’s recommendations (Roche Diagnostics). These MMR IHC results were scored blinded to the original IHC result or tumor panel sequencing results (CR) and tumors were considered dMMR if loss of expression of one or more of the proteins in tumor cell nuclei was observed while lymphocytes and morphologically normal tissue retained protein expression.

## *DNA Extraction from Tumor and Blood Biospecimen*

A H&E-stained slide was assessed for each tumor to identify areas of high tumor cellularity for macrodissection. Formalin-fixed paraffin embedded (FFPE) tissue DNA was extracted from the tumor and surrounding normal tissue using the QIAamp DNA FFPE Tissue Kit (Qiagen, Hilden, Germany). DNA was extracted from peripheral blood lymphocytes using the DNeasy blood and tissue kit (Qiagen, Hilden, Germany) and sequenced as a matched germline reference.

## *Targeted Multi-Gene Panel Testing*

A multi-gene panel capturing 2.005 megabases (Mb), modified from the capture described in Zaidi *et al.* (Zaidi et al., 2020), was custom-designed for the analysis of tumor and matched blood-derived DNA. The panel comprised of the MMR and *EPCAM* genes as well as other established hereditary CRC and EC genes and consisted of:

1) 298 genes including hereditary CRC (Belhadj et al., 2020; Seifert et al., 2019; Weren et al., 2015) and EC (Spurdle et al., 2017) risk genes and genes that are frequently mutated in CRC, EC and SST individuals as identified by The Cancer Genome Atlas data (Cherniack et al., 2017; Levine, 2013; Muzny et al., 2012; North et al., 2018) (including *POLE, POLD1, MUTYH*);

2) 143 single nucleotide polymorphisms that have previously been identified as associated with risk of CRC and EC from genome-wide association studies (Huyghe et al., 2019; O’Mara et al., 2019; Schmit et al., 2018);

3) 28 microsatellite loci including the five ‘gold standard’ microsatellite instability (MSI) markers (BAT25, BAT26, NR-21, NR-24, and MONO-27), which are used in routine MSI polymerase chain reaction (PCR) diagnostics as recommended by the National Cancer Institute (Boland et al., 1998), to determine the MSI status in affected individuals;

4) 212 homopolymer regions distributed throughout the genome which are common targets of MSI, included to aid in determining dMMR cancers (Kim et al., 2013) and;

5) 56 structural variants known to be susceptible to copy number changes in CRCs (Peltomäki, 2001; Xie et al., 2012).

Library preparation was performed using the SureSelect^XT^ Low Input Target Enrichment System (Agilent Technologies, Santa Clara, USA) and sequenced on a single lane of a NovaSeq 6000 SP flow cell (Illumina, San Diego, United States) with 300 cycles (150 bp) paired-end reads) at the Australian Genome Research Facility. The median on-target coverage for the 134 test samples was 323.7 for the tumor DNA and 137.4 for blood-derived DNA samples, with an interquartile range of 111.8 – 426.4 and 100.6 – 204.9, respectively. The median on-target coverage for the 53 reference samples was 919.3 (interquartile range 694.6 – 1164.9) for tumor DNA samples and 160.6 (135.8 – 178.0) for blood-derived DNA samples.

## *Bioinformatic Analysis*

Adapter sequences were trimmed from raw FASTQ files using Trimmomatic (v.0.38) (Bolger et al., 2014) and aligned to the GRCh37 human reference genome using Burrows-Wheeler Aligner (v.0.7.12) to generate BAM files. Germline and somatic single-nucleotide variants (SNVs) and INDELs were called using Strelka (v.2.9.2, Illumina, San Diego, USA) using the recommended workflow (Saunders et al., 2012). Loss of heterozygosity (LOH) over the MMR, *POLD1* and *POLE* genes were called using the “LOHdeTerminator” (v.0.6, <https://github.com/supernifty/LOHdeTerminator>) and manually confirmed in BAM files using the Integrative Genomics Viewer (v.2.3). Variants were filtered for PASS with a minimum variant allele fraction of 0.1 and 50x minimum coverage. All variants of interest were verified by manual assessments.

Identified somatic single nucleotide variants (SNVs) and small insertions / deletions (INDELs) were used to calculate TMS according to the simulated annealing approach described by Huang *et al.* (Huang et al., 2018). Single Base Substitutions (TMS SBS) and ID signatures from COSMIC (v.3.2, <https://cancer.sanger.ac.uk/signatures/>, last accessed date: June 15^th^, 2022) (Tate et al., 2019) were calculated following previous publications (Alexandrov et al., 2013, 2020; Alexandrov & Stratton, 2014).

Tumor mutation burden (TMB) defined as the combined number of somatic SNVs and small insertions / deletions (INDELs) mutations per Mb was calculated as the number of PASS variants as called by Strelka where ≥10 mutations/Mb was considered hypermutated and ≥100 mutations/Mb considered ultra-hypermutated as previously determined (Campbell et al., 2017).

Details of the somatic pipeline and mutational signature calculation are available at <https://github.com/supernifty/somatic_pipeline> (v.0.3) and <https://github.com/supernifty/mutational_signature> (v.0.8), respectively. Single Base Substitutions (SBS) and small (1 to 50 bp) Insertions and Deletions (ID) TMS as described in COSMIC (v.3.2, <https://cancer.sanger.ac.uk/signatures/>, last accessed date: June 15^th^, 2022) (Tate et al., 2019) were calculated using the simulated annealing approach described by Huang *et al.* (Huang et al., 2018).

Variants were published in the Variant Call Format according to HGVS nomenclature guidelines (den Dunnen et al., 2016) using canonical RefSeq transcripts GRCh37/hg19 as determined by the Ensembl Variant Effect Predictor (McLaren et al., 2016). Following RefSeq transcripts were used for the candidate genes: *MLH1*: NM_000249.3, *MSH2*: NM_000251.2, *MSH6*: NM_000179.2, *PMS2*: NM_000535.5 and *POLD1*: NM_002691.4. For *BRAF, MUTYH* and *POLE* genes, we used following exceptions as these are the more commonly used transcripts: *BRAF:* NM_004333.4, *MUTYH:* NM_001128425.1 and *POLE:* NM_002692.4.

## *Determination of DNA Mismatch Repair Status Using the Additive Feature Combination Approach*

Overall tumor dMMR status was determined by applying the additive feature combination approach described in Walker *et al.*, (Walker et al., 2022), where ≥3 features with positivity for dMMR out of the six assessed tools/features, namely MSMuTect (Maruvka et al., 2017), MANTIS (Kautto et al., 2017), MSIseq (Ni Huang et al., 2015), MSISensor (Niu et al., 2014), INDEL count and TMS ID2+ID7 (Georgeson et al., 2021) was considered dMMR. A statistical analysis was performed to determine the optimal thresholds for differentiating dMMR from pMMR tumor status for each tumor type as reported previously (Walker et al., 2022). The thresholds or optimal cut-offs for each of the six tools/features that comprise the additive feature combination approach are shown in the table below (**Supplementary Table S1**).

## *Categorization of SLS Cases Using the Accumulated Molecular Results*

The SLS cases were categorized using the results from tumor sequencing as well as re-testing of *MLH1* methylation and MMR IHC as follows:

1) dMMR with a germline pathogenic variant identified (as determined by ClinVar, <https://www.ncbi.nlm.nih.gov/clinvar/>, last accessed date: December 7^th^, 2022) (Lynch syndrome, “dMMR-LS”),

2) dMMR with tumor *MLH1* methylation (*MLH1* methylated, “dMMR-MLH1me”),

3) dMMR with tumor and blood *MLH1* methylation (primary epimutation, “dMMR-PriEpi”),

4) dMMR with double somatic MMR variants in the same MMR gene (double somatic mutation, “dMMR-DS”),

5) dMMR with a single somatic MMR variant (single somatic mutation, “dMMR-SS”),

6) dMMR with no germline or somatic variants (suspected Lynch syndrome, “dMMR-SLS”) and

7) pMMR tumors with neither germline or somatic mutations nor hypermethylation of the *MLH1* gene (DNA mismatch repair proficient, “pMMR”).

## *PREMM5: Lynch Syndrome Prediction Model*

The PREMM5 scores, clinically used to predict the likelihood for Lynch syndrome in patients of interest, were calculated using the revised model as described by Kastrinos *et al.* (Kastrinos et al., 2017) and implemented using their website (<https://premm.dfci.harvard.edu/>, last accessed date: 13^th^ November, 2022). A recommended cut-off of ≥2.5% was used to predict Lynch syndrome (Kastrinos et al., 2017).

## *References*

Alexandrov, L. B., Kim, J., Haradhvala, N. J., Huang, M. N., Tian Ng, A. W., Wu, Y., Boot, A., Covington, K. R., Gordenin, D. A., Bergstrom, E. N., Islam, S. M. A., Lopez-Bigas, N., Klimczak, L. J., McPherson, J. R., Morganella, S., Sabarinathan, R., Wheeler, D. A., Mustonen, V., Getz, G., … Stratton, M. R. (2020). The repertoire of mutational signatures in human cancer. *Nature*, *578*(7793), Article 7793. https://doi.org/10.1038/s41586-020-1943-3

Alexandrov, L. B., Nik-Zainal, S., Wedge, D. C., Aparicio, S. A. J. R., Behjati, S., Biankin, A. V., Bignell, G. R., Bolli, N., Borg, A., Børresen-Dale, A.-L., Boyault, S., Burkhardt, B., Butler, A. P., Caldas, C., Davies, H. R., Desmedt, C., Eils, R., Eyfjörd, J. E., Foekens, J. A., … Stratton, M. R. (2013). Signatures of mutational processes in human cancer. *Nature*, *500*(7463), Article 7463. https://doi.org/10.1038/nature12477

Alexandrov, L. B., & Stratton, M. R. (2014). Mutational signatures: The patterns of somatic mutations hidden in cancer genomes. *Current Opinion in Genetics & Development*, *24*, 52–60. https://doi.org/10.1016/j.gde.2013.11.014

Belhadj, S., Terradas, M., Munoz-Torres, P. M., Aiza, G., Navarro, M., Capellá, G., & Valle, L. (2020). Candidate genes for hereditary colorectal cancer: Mutational screening and systematic review. *Human Mutation*, *41*(9), 1563–1576. https://doi.org/10.1002/humu.24057

Boland, C. R., Thibodeau, S. N., Hamilton, S. R., Sidransky, D., Eshleman, J. R., Burt, R. W., Meltzer, S. J., Rodriguez-Bigas, M. A., Fodde, R., Ranzani, G. N., & Srivastava, S. (1998). A National Cancer Institute Workshop on Microsatellite Instability for cancer detection and familial predisposition: Development of international criteria for the determination of microsatellite instability in colorectal cancer. *Cancer Res*, *58*(22), 5248–5257.

Bolger, A. M., Lohse, M., & Usadel, B. (2014). Trimmomatic: A flexible trimmer for Illumina sequence data. *Bioinformatics (Oxford, England)*, *30*(15), 2114–2120. https://doi.org/10.1093/bioinformatics/btu170

Campbell, B. B., Light, N., Fabrizio, D., Zatzman, M., Fuligni, F., de Borja, R., Davidson, S., Edwards, M., Elvin, J. A., Hodel, K. P., Zahurancik, W. J., Suo, Z., Lipman, T., Wimmer, K., Kratz, C. P., Bowers, D. C., Laetsch, T. W., Dunn, G. P., Johanns, T. M., … Shlien, A. (2017). Comprehensive Analysis of Hypermutation in Human Cancer. *Cell*, *171*(5), 1042-1056.e10. https://doi.org/10.1016/j.cell.2017.09.048

Cherniack, A. D., Shen, H., Walter, V., Stewart, C., Murray, B. A., Bowlby, R., Hu, X., Ling, S., Soslow, R. A., Broaddus, R. R., Zuna, R. E., Robertson, G., Laird, P. W., Kucherlapati, R., Mills, G. B., Akbani, R., Ally, A., Auman, J. T., Balasundaram, M., … Levine, D. A. (2017). Integrated Molecular Characterization of Uterine Carcinosarcoma. *Cancer Cell*, *31*(3), 411–423. https://doi.org/10.1016/j.ccell.2017.02.010

den Dunnen, J. T., Dalgleish, R., Maglott, D. R., Hart, R. K., Greenblatt, M. S., McGowan-Jordan, J., Roux, A.-F., Smith, T., Antonarakis, S. E., & Taschner, P. E. M. (2016). HGVS Recommendations for the Description of Sequence Variants: 2016 Update. *Human Mutation*, *37*(6), 564–569. https://doi.org/10.1002/humu.22981

Georgeson, P., Pope, B. J., Rosty, C., Clendenning, M., Mahmood, K., Joo, J. E., Walker, R., Hutchinson, R. A., Preston, S., Como, J., Joseland, S., Win, A. K., Macrae, F. A., Hopper, J. L., Mouradov, D., Gibbs, P., Sieber, O. M., O’Sullivan, D. E., Brenner, D. R., … Buchanan, D. D. (2021). Evaluating the utility of tumour mutational signatures for identifying hereditary colorectal cancer and polyposis syndrome carriers. *Gut*, *70*(11), 2138–2149. https://doi.org/10.1136/gutjnl-2019-320462

Huang, X., Wojtowicz, D., & Przytycka, T. M. (2018). Detecting presence of mutational signatures in cancer with confidence. *Bioinformatics (Oxford, England)*, *34*(2), 330–337. https://doi.org/10.1093/bioinformatics/btx604

Huyghe, J. R., Bien, S. A., Harrison, T. A., Kang, H. M., Chen, S., Schmit, S. L., Conti, D. V., Qu, C., Jeon, J., Edlund, C. K., Greenside, P., Wainberg, M., Schumacher, F. R., Smith, J. D., Levine, D. M., Nelson, S. C., Sinnott-Armstrong, N. A., Albanes, D., Alonso, M. H., … Peters, U. (2019). Discovery of common and rare genetic risk variants for colorectal cancer. *Nature Genetics*, *51*(1), 76–87. https://doi.org/10.1038/s41588-018-0286-6

Kastrinos, F., Uno, H., Ukaegbu, C., Alvero, C., McFarland, A., Yurgelun, M. B., Kulke, M. H., Schrag, D., Meyerhardt, J. A., Fuchs, C. S., Mayer, R. J., Ng, K., Steyerberg, E. W., & Syngal, S. (2017). Development and Validation of the PREMM5 Model for Comprehensive Risk Assessment of Lynch Syndrome. *Journal of Clinical Oncology: Official Journal of the American Society of Clinical Oncology*, *35*(19), 2165–2172. https://doi.org/10.1200/JCO.2016.69.6120

Kautto, E. A., Bonneville, R., Miya, J., Yu, L., Krook, M. A., Reeser, J. W., & Roychowdhury, S. (2017). Performance evaluation for rapid detection of pan-cancer microsatellite instability with MANTIS. *Oncotarget*, *8*(5), 7452–7463. https://doi.org/10.18632/oncotarget.13918

Kim, T.-M., Laird, P. W., & Park, P. J. (2013). The Landscape of Microsatellite Instability in Colorectal and Endometrial Cancer Genomes. *Cell*, *155*(4), 858–868. https://doi.org/10.1016/j.cell.2013.10.015

Levine, D. A. (2013). Integrated genomic characterization of endometrial carcinoma. *Nature*, *497*(7447), Article 7447. https://doi.org/10.1038/nature12113

Maruvka, Y. E., Mouw, K. W., Karlic, R., Parasuraman, P., Kamburov, A., Polak, P., Haradhvala, N. J., Hess, J. M., Rheinbay, E., Brody, Y., Koren, A., Braunstein, L. Z., D’Andrea, A., Lawrence, M. S., Bass, A., Bernards, A., Michor, F., & Getz, G. (2017). Analysis of somatic microsatellite indels identifies driver events in human tumors. *Nature Biotechnology*, *35*(10), Article 10. https://doi.org/10.1038/nbt.3966

McLaren, W., Gil, L., Hunt, S. E., Riat, H. S., Ritchie, G. R. S., Thormann, A., Flicek, P., & Cunningham, F. (2016). The Ensembl Variant Effect Predictor. *Genome Biology*, *17*(1), 122. https://doi.org/10.1186/s13059-016-0974-4

Muzny, D. M., Bainbridge, M. N., Chang, K., Dinh, H. H., Drummond, J. A., Fowler, G., Kovar, C. L., Lewis, L. R., Morgan, M. B., Newsham, I. F., Reid, J. G., Santibanez, J., Shinbrot, E., Trevino, L. R., Wu, Y.-Q., Wang, M., Gunaratne, P., Donehower, L. A., Creighton, C. J., … Tissue source sites and disease working group. (2012). Comprehensive molecular characterization of human colon and rectal cancer. *Nature*, *487*(7407), Article 7407. https://doi.org/10.1038/nature11252

Ni Huang, M., McPherson, J. R., Cutcutache, I., Teh, B. T., Tan, P., & Rozen, S. G. (2015). MSIseq: Software for Assessing Microsatellite Instability from Catalogs of Somatic Mutations. *Scientific Reports*, *5*(1), Article 1. https://doi.org/10.1038/srep13321

Niu, B., Ye, K., Zhang, Q., Lu, C., Xie, M., McLellan, M. D., Wendl, M. C., & Ding, L. (2014). MSIsensor: Microsatellite instability detection using paired tumor-normal sequence data. *Bioinformatics (Oxford, England)*, *30*(7), 1015–1016. https://doi.org/10.1093/bioinformatics/btt755

North, J. P., Golovato, J., Vaske, C. J., Sanborn, J. Z., Nguyen, A., Wu, W., Goode, B., Stevers, M., McMullen, K., Perez White, B. E., Collisson, E. A., Bloomer, M., Solomon, D. A., Benz, S. C., & Cho, R. J. (2018). Cell of origin and mutation pattern define three clinically distinct classes of sebaceous carcinoma. *Nature Communications*, *9*(1), Article 1. https://doi.org/10.1038/s41467-018-04008-y

O’Mara, T. A., Glubb, D. M., Kho, P. F., Thompson, D. J., & Spurdle, A. B. (2019). Genome-Wide Association Studies of Endometrial Cancer: Latest Developments and Future Directions. *Cancer Epidemiology, Biomarkers & Prevention: A Publication of the American Association for Cancer Research, Cosponsored by the American Society of Preventive Oncology*, *28*(7), 1095–1102. https://doi.org/10.1158/1055-9965.EPI-18-1031

Pecorelli, S. (2009). Revised FIGO staging for carcinoma of the vulva, cervix, and endometrium. *International Journal of Gynaecology and Obstetrics: The Official Organ of the International Federation of Gynaecology and Obstetrics*, *105*(2), 103–104. https://doi.org/10.1016/j.ijgo.2009.02.012

Peltomäki, P. (2001). Deficient DNA mismatch repair: A common etiologic factor for colon cancer. *Human Molecular Genetics*, *10*(7), 735–740. https://doi.org/10.1093/hmg/10.7.735

Saunders, C. T., Wong, W. S. W., Swamy, S., Becq, J., Murray, L. J., & Cheetham, R. K. (2012). Strelka: Accurate somatic small-variant calling from sequenced tumor–normal sample pairs. *Bioinformatics*, *28*(14), 1811–1817. https://doi.org/10.1093/bioinformatics/bts271

Schmit, S. L., Edlund, C. K., Schumacher, F. R., Gong, J., Harrison, T. A., Huyghe, J. R., Qu, C., Melas, M., Van Den Berg, D. J., Wang, H., Tring, S., Plummer, S. J., Albanes, D., Alonso, M. H., Amos, C. I., Anton, K., Aragaki, A. K., Arndt, V., Barry, E. L., … Gruber, S. B. (2018). Novel Common Genetic Susceptibility Loci for Colorectal Cancer. *JNCI Journal of the National Cancer Institute*, *111*(2), 146–157. https://doi.org/10.1093/jnci/djy099

Seifert, B. A., McGlaughon, J. L., Jackson, S. A., Ritter, D. I., Roberts, M. E., Schmidt, R. J., Thompson, B. A., Jimenez, S., Trapp, M., Lee, K., Plon, S. E., Offit, K., Stadler, Z. K., Zhang, L., Greenblatt, M. S., & Ferber, M. J. (2019). Determining the clinical validity of hereditary colorectal cancer and polyposis susceptibility genes using the Clinical Genome Resource Clinical Validity Framework. *Genetics in Medicine: Official Journal of the American College of Medical Genetics*, *21*(7), 1507–1516. https://doi.org/10.1038/s41436-018-0373-1

Spurdle, A. B., Bowman, M. A., Shamsani, J., & Kirk, J. (2017). Endometrial cancer gene panels: Clinical diagnostic vs research germline DNA testing. *Modern Pathology*, *30*(8), Article 8. https://doi.org/10.1038/modpathol.2017.20

Tate, J. G., Bamford, S., Jubb, H. C., Sondka, Z., Beare, D. M., Bindal, N., Boutselakis, H., Cole, C. G., Creatore, C., Dawson, E., Fish, P., Harsha, B., Hathaway, C., Jupe, S. C., Kok, C. Y., Noble, K., Ponting, L., Ramshaw, C. C., Rye, C. E., … Forbes, S. A. (2019). COSMIC: The Catalogue Of Somatic Mutations In Cancer. *Nucleic Acids Research*, *47*(D1), D941–D947. https://doi.org/10.1093/nar/gky1015

Walker, R., Georgeson, P., Mahmood, K., Jihoon, E. J., Makalic, E., Clendenning, M., Como, J., Preston, S., Joseland, S., Pope, B. J., Hutchinson, R. A., Kasem, K., Walsh, M., Macrae, F. A., Win, A. K., Hopper, J. L., Mouradov, D., Gibbs, P., Sieber, O. M., … Buchanan, D. D. (2022). Evaluating multiple next-generation sequencing derived tumor features to accurately predict DNA mismatch repair status. *The Journal of Molecular Diagnostics: JMD*.

Weren, R. D. A., Ligtenberg, M. J. L., Kets, C. M., de Voer, R. M., Verwiel, E. T. P., Spruijt, L., van Zelst-Stams, W. A. G., Jongmans, M. C., Gilissen, C., Hehir-Kwa, J. Y., Hoischen, A., Shendure, J., Boyle, E. A., Kamping, E. J., Nagtegaal, I. D., Tops, B. B. J., Nagengast, F. M., Geurts van Kessel, A., van Krieken, J. H. J. M., … Hoogerbrugge, N. (2015). A germline homozygous mutation in the base-excision repair gene NTHL1 causes adenomatous polyposis and colorectal cancer. *Nature Genetics*, *47*(6), Article 6. https://doi.org/10.1038/ng.3287

Xie, T., D’ Ario, G., Lamb, J. R., Martin, E., Wang, K., Tejpar, S., Delorenzi, M., Bosman, F. T., Roth, A. D., Yan, P., Bougel, S., Di Narzo, A. F., Popovici, V., Budinská, E., Mao, M., Weinrich, S. L., Rejto, P. A., & Hodgson, J. G. (2012). A comprehensive characterization of genome-wide copy number aberrations in colorectal cancer reveals novel oncogenes and patterns of alterations. *PloS One*, *7*(7), e42001. https://doi.org/10.1371/journal.pone.0042001

Zaidi, S. H., Harrison, T. A., Phipps, A. I., Steinfelder, R., Trinh, Q. M., Qu, C., Banbury, B. L., Georgeson, P., Grasso, C. S., Giannakis, M., Adams, J. B., Alwers, E., Amitay, E. L., Barfield, R. T., Berndt, S. I., Borozan, I., Brenner, H., Brezina, S., Buchanan, D. D., … Peters, U. (2020). Landscape of somatic single nucleotide variants and indels in colorectal cancer and impact on survival. *Nature Communications*, *11*(1), 3644. https://doi.org/10.1038/s41467-020-17386-z

## **SUPPLEMENTARY TABLES**

## **Table S1.** Table displaying optimal cut-offs for the six tumor features determined previously (Walker et al., 2022) in the additive feature combination approach.

Abbreviations: colorectal cancer, CRC; endometrial cancer, EC; sebaceous skin tumor, SST; DNA mismatch repair deficient, dMMR; DNA mismatch repair proficient, pMMR; insertions / deletions, INDELs; tumor mutational signatures, TMS; small (1 to 50 base pair) insertions / deletions, IDs; single base substitution, SBS and tumor mutational burden, TMB.

|  | CRC | EC | SST |
| --- | --- | --- | --- |
| Tumor Features | pMMR < n ≥ dMMR | pMMR < n ≥ dMMR | pMMR < n ≥ dMMR |
| MSMuTect | 48 | 31 | 25 |
| MSIseq | 2.494 | 0.499 | 0.499 |
| MANTIS | 0.252 | 0.274 | 0.217 |
| INDEL count | 5 | 1 | 3 |
| MSISensor | 6.9 | 21.21 | 2.05 |
| TMS ID2+ID7 | 0.504 | 0.182 | 0.547 |

## **Table S2.** SLS tumors (n=13) that showed discordant MMR IHC findings between clinical diagnostic testing before study entry and testing completed internally during this study and the change in their MMR status and/or pattern of MMR protein loss.

Abbreviations: colorectal cancer, CRC; endometrial cancer, EC; sebaceous skin tumor, SST; DNA mismatch repair, MMR; DNA mismatch repair deficient, dMMR; DNA mismatch repair proficient, pMMR; not tested, NT. + Indicates heterogeneous loss of MMR protein expression by IHC.

| *^+^ Indicates heterogeneous loss of MMR protein expression by IHC.* |
| --- |
| *^a^ All pre-study clinical MLH1 methylation results were generated using methylation specific - multiplexed ligation dependent probe amplification assay (MS-MLPA), unless where indicated with BRAF V600E testing.* |
| *^b^ Only MLH1 protein expression was tested due to only a single slide available for IHC.* |

## **Table S3.** The concordance between the final MMR IHC result and the predicted dMMR status from the additive feature combination approach overall and by tumor type.

Abbreviations: colorectal cancer, CRC; endometrial cancer, EC; sebaceous skin tumor, SST; DNA mismatch repair deficient, dMMR; DNA mismatch repair proficient, pMMR.

## **Table S4.** The tumor *MLH1* methylation testing completed for SLS tumors prior to entering the study showing either negative, inconclusive, or not tested results and the subsequent *MLH1* methylation testing results from internal testing using MethyLight and MS-HRM assays highlighting the positive *MLH1* methylation results found by this study.

Abbreviations: colorectal cancer, CRC; endometrial cancer, EC; sebaceous skin tumor, SST; DNA mismatch repair, MMR; DNA mismatch repair deficient, dMMR; DNA mismatch repair proficient, pMMR; BRAF V600E, BRAF; not tested, NT; not applicable, NA. + Indicates heterogeneous loss of MMR protein expression by IHC.

**Table S4 continued**

| *^+^ Indicates heterogeneous loss of MMR protein expression by IHC.* |
| --- |
| *^a^ All clinical MLH1 methylation results were generated using methylation specific - multiplexed ligation dependent probe amplification assay (MS-MLPA), unless where indicated that BRAF V600E testing was used as a surrogate for MLH1 methylation testing.* |
| *^b^ Clinics indicated DNA quality as low.* |
| *^c^ Blood DNA was not tested for MLH1 methylation.* |
| *^d^ In-house testing showed low levels of MLH1 methylation but called positive overall.* |
| *^e^ Blood-derived DNA was tested for MLH1 methylation and found to be negative for MLH1 methylation.* |
| *^f^ Repeated twice.* |
| *^g^ Only tested on blood-derived DNA, no tumor testing was performed.* |

## **Table S5.** Presentation of germline pathogenic variants and variants of uncertain clinical significance (VUS) identified in the MMR, *MUTYH* and *POLE* genes.

Abbreviations: colorectal cancer, CRC; endometrial cancer, EC; sebaceous skin tumor, SST; DNA mismatch repair, MMR; DNA mismatch repair deficient, dMMR; DNA mismatch repair proficient, pMMR; loss of heterozygosity, LOH; tumor mutational signature, TMS; variant of uncertain clinical significance, VUS; Lynch syndrome, LS; *MLH1* methylation, dMMR-MLH1me; double somatic mutation, dMMR-DS.

| No. | ID | Tumor Type | Sex | AgeDx | **FINAL MMR IHC Status** | MMR Status by Additive Feature Approach | **Germline Variant** | VarSome ACMG Implementation for Germline Variant | New Proposed Variant Classification | Somatic MMR Events | **FINAL Tumor / Individual Categorization** |
| --- | --- | --- | --- | --- | --- | --- | --- | --- | --- | --- | --- |
| 1 | SLS135 | CRC | M | 24 | **Loss of all four MMR proteins** | dMMR (3/6) | ***MSH2*: deletion of exon 7** | - | PATHOGENIC | Somatic mutation (2nd hit) *MSH2*: c.2458+1G>A p.? | **dMMR - LS + (dMMR - MLH1me)** |
| 2 | SLS256 | EC | F | 59 | **MLH1/PMS2 and MSH6** | pMMR (2/6) | ***MSH6*: c.3834_3849dup p.Thr1284Glnfs*10** | Pathogenic (Class 5): PVS1 \| PM2 \| PP3 | PATHOGENIC | Somatic mutation (2nd hit) *MSH6*: c.3261del p.Phe1088Serfs*2 | **dMMR - LS + (dMMR - MLH1me)** |
| 3 | SLS019 | SST | M | 72 | **MLH1/PMS2** | dMMR (6/6) | ***MLH1*: c.400A>G p.Lys134Glu** | Uncertain Significance (Class 3): PM1 \| PP3 \| PM2 | VUS | LOH across *MLH1* \| somatic mutation *MLH1*: c.199G>A p.Gly67Arg | **dMMR - DS (MLH1)** |
| 4 | SLS328 | CRC | F | 41 | **MSH2/MSH6** | dMMR (6/6) | ***MLH1*: c.1153C>T p.Arg385Cys** | Uncertain Significance (Class 3): PP5 \| PM2 \| PP2 \| PP3 | VUS | No somatic mutations or LOH across *MLH1* | **dMMR - DS (MSH2)** |
| 5 | SLS395 | CRC | F | 38 | **MLH1/PMS2** | dMMR (6/6) | ***MSH2*: c.668T>C p.Leu223Pro** | Uncertain Significance (Class 3): PP3 \| PM2 | VUS | No somatic mutations or LOH across *MSH2* | **dMMR - DS (MLH1)** |
| 6 | SLS089 | CRC | F | 42 | **MLH1/PMS2** | dMMR (6/6) | ***MSH6*: c.2827G>T p.Asp943Tyr** | Uncertain Significance (Class 3): PM2 \| PP3 | VUS | No somatic mutations or LOH across *MSH6* | **dMMR - DS (MLH1)** |
| 7 | SLS152 | CRC | F | 40 | **MLH1/PMS2** | dMMR (5/6) | ***MSH6*: c.2963G>A p.Arg988His** | Uncertain Significance (Class 3): PM2 \| BP4 | VUS | Somatic mutation in *MSH6*: c.2963G>A p.Arg988His | **dMMR - MLH1me** |
| 8 | SLS263 | EC | F | 62 | **MLH1/PMS2** | dMMR (5/6) | ***MSH6*: c.*85T>A p.?** | Uncertain significance (Class 3): PM2 \| BP4 | VUS | Somatic mutation in *MSH6*: c.3261del p.Phe1088Serfs*2 | **dMMR - DS (MLH1)** |
| 9 | SLS266 | SST | M | 69 | **MSH2/MSH6** | dMMR (6/6) | ***PMS2*: c.*3G>A p.?** | Uncertain Significance (Class 3): PM2 \| BP4 | VUS | No somatic mutations or LOH across *PMS2* | **dMMR - DS (MSH2)** |
| 10 | SLS104 | SST | M | 71 | **MSH2/MSH6** | dMMR (5/6) | ***MSH2*: c.138C>G p.His46Gln** | Uncertain Significance (Class 3): PS1 \| PM2 \| PP2 \| PP3 \| PP5 \| BP6 | LIKELY BENIGN | LOH across *MSH2* \| MSH2: c.1759G>C p.Gly587Arg | **dMMR - DS (MSH2)** |
| 11 | SLS202 | EC | F | 65 | **MSH6** | dMMR (3/6) | ***MSH2*: c.138C>G p.His46Gln** | Uncertain Significance (Class 3): PS1 \| PM2 \| PP2 \| PP3 \| PP5 \| BP6 | LIKELY BENIGN | Somatic mutation in *MSH2*: c.1354G>A p.Glu452Lys | **dMMR - DS (MSH6)** |
| 12 | SLS202 | EC | F | 65 | **MSH6** | dMMR (3/6) | ***MUTYH*: c.536A>G p.Tyr179Cys** | Pathogenic (Class 5): PP5 \| PS3 \| PM2 | PATHOGENIC | No *MUTYH* deficiency TMS (SBS18 / SBS36), no somatic mutations or LOH across *MUTYH* | **dMMR - DS (MSH6)** |
| 13 | SLS267 | EC | F | 64 | **MSH2/MSH6** | dMMR (5/6) | ***MUTYH*: c.1187G>A p.Gly396Asp** | Pathogenic (Class 5): PS3 \| PP3 \| PP5 \| PM5 | PATHOGENIC | No *MUTYH* deficiency TMS (SBS18 / SBS36) , no somatic mutations or LOH across *MUTYH* | **dMMR - DS (MSH2)** |
| 14 | SLS006 | SST | M | 75 | **MSH2/MSH6** | dMMR (6/6) | ***MUTYH*: c.1187G>A p.Gly396Asp** | Pathogenic (Class 5): PS3 \| PP3 \| PP5 \| PM5 | PATHOGENIC | No *MUTYH* deficiency TMS (SBS18 / SBS36) , no somatic mutations or LOH across *MUTYH* | **dMMR - DS (MSH2)** |
| 15 | SLS092 | CRC | M | 72 | **Normal** | pMMR (0/6) | ***MUTYH*: c.1276C>T p.Arg426Cys** | Likely Benign (Class 2): BP4 \| PM2 | VUS | No *MUTYH* deficiency TMS (SBS18 / SBS36) , no somatic mutations or LOH across *MUTYH* | **pMMR** |
| 16 | SLS236 | CRC | M | 34 | **PMS2** | dMMR (6/6) | ***POLE:* c.825C>G p.Asp275Glu** | Uncertain Significance (Class 3): PM2 \| PP3 \| BP1 | VUS | No *POLE/POLD1* related TMS; Somatic mutation *POLD1*: c.1327C>T p.Arg443Trp; ultra-hypermutated TMB (437 mutations/Mb) | **dMMR - DS (PMS2)** |
| 17 | SLS239 | CRC | M | 60 | **MSH2/MSH6** | dMMR (6/6) | ***POLE*: c.861T>A p.Asp287Glu** | Uncertain Significance (Class 3): PM2 \| PP3\| BP1 | VUS | No *POLE/POLD1* related TMS; somatic *POLD1* mutation c.1054C>T p.Arg352Cys; ultra-hypermutated TMB (118 mutations/Mb) | **dMMR - DS (MSH2)** |

## **Table S6.** Summary of the clinicopathological features for the double somatic MMR mutation (dMMR-DS) tumors overall and by tumor type.

Summary of the clinicopathological features for the double somatic MMR mutation (dMMR-DS) tumors overall and by tumor type.

Abbreviations: Standard deviation, SD; colorectal cancer, CRC; endometrial cancer, EC; sebaceous skin tumor, SST; DNA mismatch repair, MMR; DNA mismatch repair deficient, dMMR; DNA mismatch repair deficient tumor with double somatic mutations, dMMR-DS.

## **SUPPLEMENTARY FIGURES**

## **Figure S1. Bar plots presenting the results from the additive tumor feature combination approach to assess the MMR status in the double somatic mutation cohort for A) all tumors combined and separated by B) CRC, C) EC and D) SST tissue types.**

Abbreviations: DNA mismatch repair, MMR; colorectal cancer, CRC; endometrial cancer, EC; sebaceous skin tumor, SST; double somatic mutation, dMMR-DS; positive *MLH1* methylation, dMMR-MLH1me; Lynch syndrome, dMMR-LS; primary epimutation, dMMR-PriEpi; DNA mismatch repair proficient, pMMR; single somatic mutation, dMMR-SS; suspected Lynch syndrome, dMMR-SLS.

## **Figure S2: Bar plot presenting the prevalence of pathogenic / likely pathogenic somatic mutations (including loss of heterozygosity, LOH) by subtype for the study cohort.**

Excluded tumors that presented with loss of all four MMR proteins (n=2) and MLH1/PMS2 with concurrent MSH6 loss (n=2) by MMR IHC. Number of somatic events were counted fitting to observed loss in MMR IHC. For pMMR, somatic events observed in all four MMR genes were added to somatic variant count. Excluded tumors that presented with loss of all four MMR proteins (n = 2) and MLH1/PMS2 with concurrent MSH6 loss (n = 1) by MMR IHC. Number of somatic events were counted fitting to observed loss in MMR IHC. For pMMRs, somatic events in all four MMR variants were added to the somatic variant count***.*** Abbreviations: DNA mismatch repair, MMR; immunohistochemistry, IHC.

## **Figure S3: Pie graphs displaying the frequency of the mutation combination type (two single somatic mutations versus a single somatic mutation with loss of heterozygosity (LOH)) as well as the type of mutation A) overall and B) separated by tissue type.**

Abbreviations: Loss of heterozygosity, LOH; colorectal cancer, CRC; endometrial cancer, EC; sebaceous skin tumor, SST.

## **Figure S4: Bar graphs presenting the site distribution in the double somatic mutation cohort across all CRCs and SSTs.**

Abbreviations: Colorectal cancer, CRC; sebaceous skin tumor, SST; double somatic mutations, dMMR-DS; positive *MLH1* methylation, dMMR-MLH1me; Lynch syndrome, dMMR-LS; primary *MLH1* epimutation, dMMR-PriEpi; DNA mismatch repair proficient, pMMR; single somatic mutation, dMMR-SS; suspected Lynch syndrome, dMMR-SLS.

## **Figure S5: Boxplots presenting the site distribution in the double somatic mutation cohort across all A) CRCs and B) SSTs. Significant (< 0.05) p-values are indicated for pairwise (t-test) and multigroup comparisons (Anova).**

Abbreviations: Colorectal cancer, CRC; sebaceous skin tumor, SST; double somatic mutations, DS.

## **Figure S6: Scatter plots presenting the PREMM5 score distribution in the test cohort for A) all tumors combined and separated by B) CRC, C) EC and D) SST tissue types.**

Abbreviations: Colorectal cancer, CRC; endometrial cancer, EC; sebaceous skin tumor, SST; double somatic mutations, dMMR-DS; positive *MLH1* methylation, dMMR-MLH1me; Lynch syndrome, dMMR-LS; primary *MLH1* epimutation, dMMR-PriEpi; single somatic mutation, dMMR-SS.


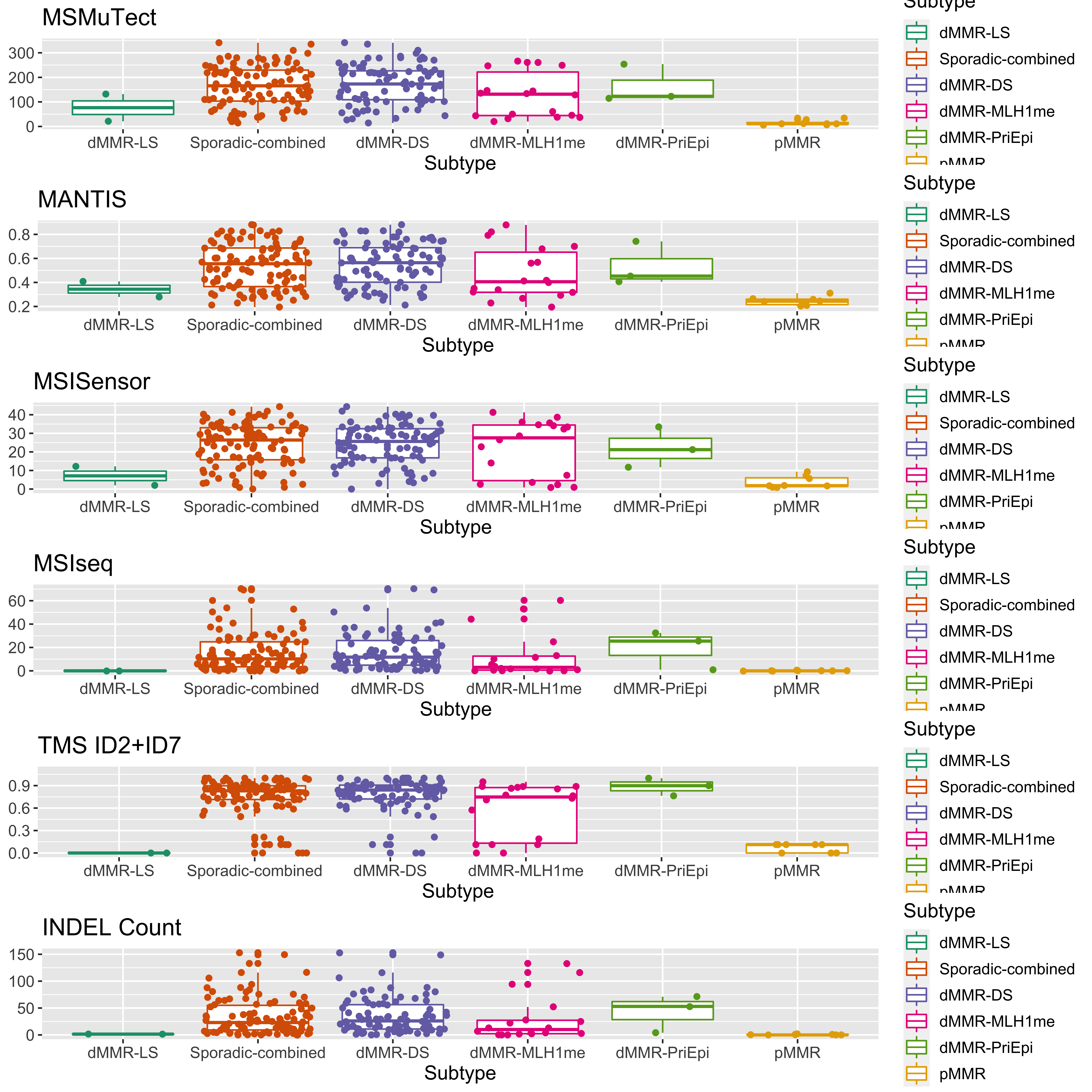




## **Figure S7: The distribution of tumor values for each of the six features that are included in the additive feature combination approach for determining tumor dMMR status grouped by molecular subtype and by combining sporadic dMMR groups dMMR-DS and dMMR-MLH1me into a “sporadic combined” group.**

Abbreviations: Lynch syndrome, dMMR-LS; double somatic mutations, dMMR-DS; positive *MLH1* methylation, dMMR-MLH1me; primary *MLH1* epimutation, dMMR-PriEpi.
